# What Governs Enzyme Activity? For One Enzyme, Charge Contributes Only Weakly

**DOI:** 10.1371/journal.pbio.0040133

**Published:** 2006-03-28

**Authors:** Richard Robinson

The sugar on your table and the oxygen in the air don't spontaneously ignite, but why not? The answer is that the conversion from reactants—sugar plus oxygen—to products—carbon dioxide plus water—requires the reactants to first adopt an extremely unstable configuration, called the transition state, in which their bonds are weakened, but newer, stronger ones have not yet formed. The “energy hill” that separates reactants from the transition state is just too high, so your sugar remains stable at room temperature.

Not so inside a cell, where enzymes catalyze thousands of different reactions that would take days, or millennia, without them. There, a reactant—called a substrate—fits into the enzyme's active site, a pocket or groove on its surface. The active site is lined with chemical groups whose shape and charge complement the shape and charge of the substrate, positive meeting negative, bump nestling into hole.

But while the reactant fits in nicely, much of the catalytic power of the enzyme has been thought to be derived from making an even better fit with the transition state. To do this, the enzyme first forms weak, temporary bonds with the reactant. The shape and charge of the active site are such that, as the reactant deforms into the transition-state configuration, those bonds become stronger. Thus, the enzyme can stabilize the transition state, lowering the height of the energy hill and thereby increasing the probability that the reactants will convert into products. Enzymes typically speed up a reaction by many orders of magnitude—a rate increase of a trillion-fold is routine for enzymes.

Shape and charge complementarity between enzyme and substrate have been proposed as keys to enzyme function, but are both equally important? That question is devilishly hard to answer, for the most fundamental of reasons: shape and charge are interdependent in most cases, and altering a molecule's shape (by inserting a larger atom, say) also changes its charge distribution. In a new study, Daniel Kraut, Daniel Herschlag, and colleagues separate the two effects and show that, for at least this one enzyme, charge makes only a modest contribution to catalytic power.

The enzyme ketosteroid isomerase (KSI) rearranges the bonds within its substrate, a multi-ring steroid molecule, by shifting a hydrogen ion from one carbon to another. One step in this process is the formation of two weak, temporary bonds, called hydrogen bonds, between KSI and an oxygen atom on the substrate. As the substrate deforms into the transition state, this oxygen becomes partially negatively charged, and the hydrogen bonds become stronger.

KSI can bind other molecules that fit the active site, including one called a phenolate anion. This compound has an oxygen atom in the same position as the steroid oxygen, but phenolate's oxygen is negatively charged, mimicking the transition state for the steroid. That charge can be made weaker or stronger by adding different chemical groups to the far end of the phenolate. Because these additions are made away from the active site, the shape of the molecule within the active site doesn't change, and the authors could evaluate charge independent of shape.

The authors did not measure reaction rate directly, but instead measured a key factor that determines reaction rate, the strength of binding interactions formed to the variably charged phenolate anion—a simple-enough sounding procedure that nonetheless drew on the full range of tools in the modern chemist's toolbox, from NMR spectroscopy to calorimetry to X-ray crystallography. Over the entire range of compounds tested, they found a difference in binding strength of only 1.5-fold, corresponding to an estimated change of at most 300-fold in the reaction rate. The authors propose that several other factors, including shape, each contribute modestly to catalysis.

While these results are directly applicable to only KSI, they provide a window onto the factors affecting catalysis in many other enzymes. Calculations based on these results may allow estimation of the effects of charge in other enzymes that cannot be manipulated in this same way. The complementary experiment—altering shape while keeping charge constant—may be even harder, and remains to be done.

## 

**Figure:**
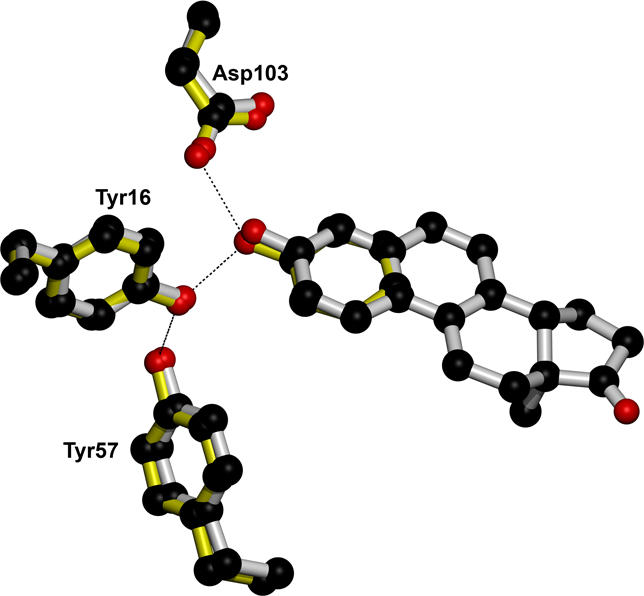
Superimposed structure of the complexes formed between the active site of ketosteroid isomerase (the three numbered amino acids to the left) and either the transition-state analog of the steroid equilenin (grey) or the phenolate anion (gold). Chemical groups can be added to the far right side of the phenolate to vary the charge on the oxygen on the left side.

